# C/EBPβ regulates multiple IL-1β-induced human astrocyte inflammatory genes

**DOI:** 10.1186/1742-2094-9-177

**Published:** 2012-07-20

**Authors:** Jerel Fields, Anuja Ghorpade

**Affiliations:** 1 University of North Texas Health Science Center, Camp Bowie Blvd, 3500, Fort Worth, TX, USA

**Keywords:** Astrocyte, Interleukin-1β, C/EBPβ, ERK1/2, p38K

## Abstract

**Background:**

CCAAT enhancer-binding protein (C/EBP)β regulates gene expression in multiple organ systems and cell types, including astrocytes in the central nervous system (CNS). Inflammatory stimuli, interleukin (IL)-1β, tumor necrosis factor-α, human immunodeficiency virus (HIV)-1 and lipopolysaccharide induce astrocyte C/EBPβ expression. C/EBPβ is detectable in brains of Alzheimer’s disease (AD), Parkinson’s disease (PD) and HIV-1-associated dementia (HAD) patients, yet little is known about how C/EBPβ contributes to astrocyte gene regulation during neuroinflammation.

**Methods:**

The expression of 92 human inflammation genes was compared between IL-1β-treated primary human astrocytes and astrocytes transfected with C/EBPβ-specific small interfering (si)RNA prior to IL-1β treatment for 12 h. Transcripts altered by > two-fold compared to control were subjected to one-way analysis of variance and Newman-Keuls post-test for multiple comparisons. Expression of two genes, cyclooxygenase-2 (COX-2) and bradykinin receptor B2 (BDKRB2) was further confirmed in additional human astrocyte donors. Astrocytes were treated with mitogen-activated protein kinase-selective inhibitors, then with IL-1β for 12 or 24 h followed by COX-2 and BDKRB2, expression analyses.

**Results:**

IL-1β altered expression of 29 of 92 human inflammation genes by at least two-fold in primary human astrocytes in 12 h. C/EBPβ knockdown affected expression of 17 out of 29 IL-1β-regulated genes by > 25%. Two genes relevant to neuroinflammation, COX-2 and BDKRB2, were robustly decreased and increased, respectively, in response to C/EBPβ knockdown, and expression was confirmed in two additional donors. COX-2 and BDKRB2 mRNA remained altered in siRNA-transfected astrocytes at 12, 24 or 72 h. Inhibiting p38 kinase (p38K) activation blocked IL-1β-induced astrocyte COX-2 mRNA and protein expression, but not IL-1β-induced astrocyte BDKRB2 expression. Inhibiting extracellular-regulated kinase (ERK)1/2 activation blocked IL-1β-induced BDKRB2 mRNA expression while increasing COX-2 expression.

**Conclusion:**

These data support an essential role for IL-1β in the CNS and identify new C/EBPβ functions in astrocytes. Additionally, this work suggests p38K and ERK1/2 pathways may regulate gene expression in a complementary manner to fine tune the IL-1β-mediated astrocyte inflammatory response. Delineating a role for C/EBPβ and other involved transcription factors in human astrocyte inflammatory response may lead to effective therapies for AD, PD, HAD and other neurological disorders.

## Background

Neuroinflammation is a contributing factor of many central nervous system (CNS) pathologies; yet the details of onset and progression remain enigmatic. Astrocytes, the most numerous cells of the CNS, contribute to homeostasis in the CNS, regulate neural signaling and maintain the blood–brain barrier (BBB) [1-3]. Accordingly, astrocytes respond to inflammatory stimuli by altering gene expression, morphology and function. Activated astrocytes undergo rapid replication, migrate to areas of insult and attempt to mitigate collateral damage by isolating the damaged area [4-6]. Previously, we reported that CCAAT enhancer-binding protein (C/EBP)-β is expressed in the brains of human immunodeficiency virus (HIV)-1-infected patients and contributes to interleukin (IL)-1β-induced tissue inhibitor metalloproteinases (TIMP)-1 expression in astrocytes [7]. In a related study, we explored the signal transduction pathways mediating IL-1β-induced astrocyte C/EBPβ and TIMP-1 expression [8]. We found that a p38 kinase (p38K)-selective inhibitor blocked IL-1β-induced astrocyte C/EBPβ expression, whereas an extracellular regulated kinase (ERK) 1/2-selective inhibitor blocked IL-1β-induced astrocyte TIMP-1 expression. In this report, we explore the role of C/EBPβ in regulating IL-1β-induced astrocyte inflammatory genes and the signal transduction pathways involved.

C/EBPβ is evolutionarily conserved among species and is expressed in multiple organ systems [9-11]. The gene is expressed as a single transcript that can be translated into three isoforms: 42 kilodalton (kDa), 40 kDa and 20 kDa [12,13]. The two large isoforms, designated liver-activating proteins, are named for their transcriptional activating properties, while the 20-kDa liver-inhibiting protein is named for its inhibitory properties [14]. It is now clear that the isoform-specific divergent roles for C/EBPβ isoforms do not completely explain their function [15,16]. In the CNS, astrocytes and microglia increase C/EBPβ expression in response to various inflammatory stimuli including IL-1β, lipopolysaccharides, tumor necrosis factor (TNF)-α and HIV-1 [7,17]. Since the discovery that C/EBPβ regulates IL-6, studies have shown that it regulates nitric oxide synthase (NOS)-2, complementary protein 3 and other important genes [15,16,18-21]. These data suggest an important role of this highly conserved transcription factor, but C/EBPβ isoform-specific activity is contextual in regard to the tissue microenvironment and cell type [15,16,19]. Given the plethora of cell-type- and ligand-dependent outcomes of C/EBPβ-mediated gene responses, a complete understanding of neuroinflammation warrants elucidating C/EBPβ function in the human astrocyte inflammatory response. Our group found that C/EBPβ is expressed in the brains of HIV-1 patients and contributes to regulation of human astrocyte TIMP-1 [7]. Overall, these data implicate C/EBPβ activity during CNS pathologies, but the extent to which the transcription factor regulates global astrocyte immune responses is unknown.

It is well established that IL-1β mediates neuroinflammation through activation of glial cells and subsequent changes in gene expression [1]. Following IL-1β-mediated activation of glial cells, nuclear C/EBPβ levels increase and affect gene transcription [7,17]. In this study, we profile the role of C/EBPβ in regulating IL-1β-mediated expression of 92 inflammatory genes in primary human astrocytes. We found that IL-1β altered expression of ~32% (29/92) mRNA transcripts tested. Furthermore, C/EBPβ regulated 59% (17/29) of these genes by increasing or decreasing transcript levels. Because of their role in neuroinflammation, two genes that were affected oppositely by C/EBPβ knockdown, cyclooxygenase (COX)-2 and bradykinin receptor b2 (BDKRB2) were chosen for further studies. ERK1/2 and p38K pathway-selective inhibitors also exhibited opposite effects on IL-1β-induced astrocyte COX-2 and BDKRB2 expression. These data illustrate the complexity of the IL-1β-mediated astrocyte inflammatory response and provide details of the regulatory mechanisms involved.

## Experimental procedures

### Isolation, cultivation and activation of human astrocytes

Human astrocytes were isolated from first- and early second-trimester (gestational weeks 12–18) aborted specimens obtained from the Birth Defects Laboratory, University of Washington, Seattle, in full compliance with the ethical guidelines of the NIH, the Universities of Washington and North Texas Health Science Center. The Birth Defects Laboratory is an NIH-funded institution with the mission of disseminating tissues for the advancement of biomedical research. Astrocytes were isolated from specimens as originally described earlier [22]. Activation of astrocytes was achieved by applying IL-1β for various time intervals. IL-1β is a prototypical inflammatory cytokine expressed during HIV-1 CNS infection, making IL-1β an excellent choice for studying human astrocyte function during neuroinflammation [23]. We empirically tested IL-1β dosage and transfection of astrocytes with C/EBPβ small interfering (si)RNA as described in Fields et al. [7]. Accordingly, these data provide relevant implications for astrocyte function in many pathologies involving neuroinflammation.

### RNA isolation and TaqMan® human inflammation array and real-time reverse transcription polymerase chain reaction (RT^2^PCR)

**Figure 1:**
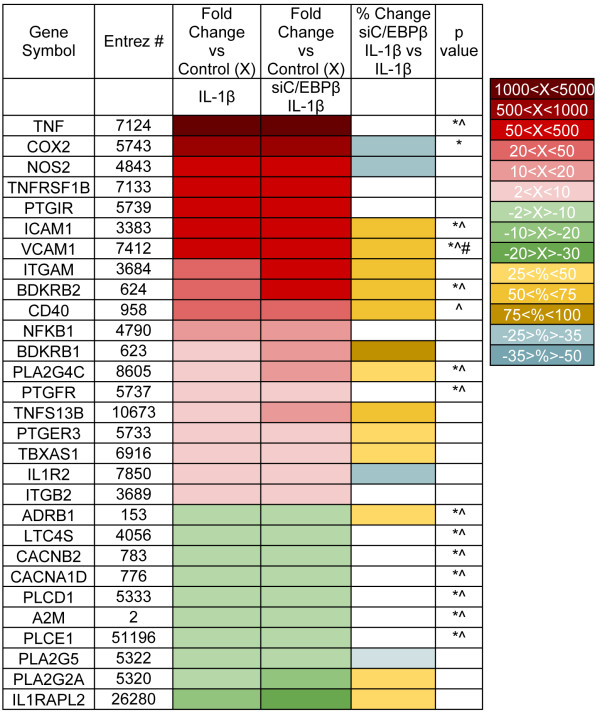
**IL-1β regulates expression of 29 of 92 human inflammation genes; C/EBPβ knockdown affects 17 of the 29 genes.** Primary human astrocytes, from two independent donors, were transfected with siCON or siC/EBPβ, cultured for 24 h and then treated with IL-1β (20 ng/ml) for 12 h. Total RNA was isolated, reverse-transcribed and then assayed for human inflammation gene expression using the TaqMan**®** Human Inflammation Assay. Data are illustrated as a heat map; red and green represent positive and negative fold-change, respectively. Increasing color contrast corresponds to increasing absolute fold-change. Orange and blue represent positive and negative percent change, respectively, in target mRNA levels in Emphasis>/Emphasis>/EBPβ + IL-1β versus IL-1β. Each individual TaqMan**®** assay of the Human Inflammation Array was independently analyzed by one-way ANOVA followed by Newman-Keuls post-test to compare among control, IL-1β and Emphasis>/Emphasis>/EBPβ treatments. Significance was set at *p* < 0.05. (Newman-Keuls post-test: control versus IL-1β, * = *p* < 0.05; control versus siC/EBPβ + IL-1β, ^ = *p* < 0.05; siC/EBPβ + IL-1β versus IL-1β, # = *p* < 0.05).

Astrocytes were transfected with nonspecific control (siCON) or C/EBPβ-specific (siC/EBPβ) siRNA by nucleofection and then cultured as adherent monolayers in 75 cm^2^ flasks at a density of 8 × 10^6^ cells per flask or six-well plates at a density of 1.6 × 10^6^ cells per well for 24 h. The following day, the medium was exchanged with or without IL-1β (20 ng/ml) for 24 h. Alternatively, astrocytes were cultured in six-well plates at a density of 1.6 × 10^6^ cells per well for 24 h, and then the medium was exchanged with or without MAPK-selective inhibitors (SB203580-sc222296 and U0126-sc202374, Santa Cruz Biotechnology, Santa Cruz, CA) for 1 h and then with inhibitor plus IL-1β (20 ng/ml) for 12 h. Astrocyte RNA was extracted (RNeasy plus mini kit, Qiagen, Alameda, CA) and reverse-transcribed into cDNA as per the manufacturer’s instructions (High Capacity cDNA Reverse Transcription Kit, Life Technologies Corp., Carlsbad, CA). The TaqMan**®** Human Inflammation Array (Life Techologies, C/N: 4414074) was used to assay for expression of 92 inflammation genes using total RNA from two independent human astrocyte donors (Figure 1). Data are illustrated in Figure 1 as a heat map; red and green represent positive and negative fold change, respectively. Increasing color intensity corresponds to increasing absolute value. Orange and blue represent positive and negative percent change, respectively, in target MRNA levels in siC/EBPβ + IL-1β versus IL-1β conditions. Readouts from each TaqMan**®** assay target were independently analyzed comparing control, IL-1β and siC/EBPβ + IL-1β by one-way analysis of variance (ANOVA) followed by Newman-Keuls post-test. Selected targets were confirmed individually by RT^2^PCR TaqMan**®** gene expression assays performed using the StepOnePlus sequence-detection system (Life Techologies), using primers specific to C/EBPβ (C/N: Hs00270923_m1), COX-2 (C/N: Hs00153133_m1), BDKRB2 (C/N: Hs00176121_m1) and glyceraldehyde phosphate dehydrogenase (GAPDH, C/N: 4310859]. The reactions were carried out at 48 °C for 30 min and 95 °C for 10 min, followed by 40 cycles of 95 °C for 15 s and 60 °C for 1 min. Samples were analyzed in triplicate. Fold changes were calculated using the comparative C_T_ method [24]. One-way ANOVA followed by Newman-Keuls post-test was used to analyze RT^2^PCR data.

### Western blot

Astrocytes were transfected with nonspecific control (siCON) or C/EBPβ-specific (siC/EBPβ) siRNA by nucleofection and then cultured as adherent monolayers in 75 cm^2^ flasks at a density of 8 × 10^6^ cells per flask. The following day, medium was exchanged with or without IL-1β (20 ng/ml) for 24 h. Alternatively, astrocytes were cultured in 75 cm^2^ flasks at a density of 8 × 10^6^ cells per flask for 24 h, and then the medium was exchanged with or without MAPK-selective inhibitors (SB203580, U0126; 20 μM) for 1 h and then with inhibitor plus IL-1β (20 ng/ml) for 24 h. Cells were lysed; total cell extracts were isolated using mammalian protein extraction reagent (Thermo Fisher Scientific, Waltham, MA, USA). Equal amounts of proteins (15 μg/lane) were resolved by 12% sodium dodecyl sulfate polyacrylamide gel electrophoresis (SDS-PAGE) and subsequently transferred to a polyvinyl difluoride (PVDF) membrane using i-Blot (Life Techologies). The membrane was incubated in anti-C/EBPβ (C-19, Santa Cruz Biotechnology) or anti-COX-2 (no. 4842, Cell Signaling Inc., Boston, MA, USA) at a dilution of 1:200. Blots were then incubated in secondary antibody (1:5,000, Promega Inc., Madison, WI). β-actin (Sigma-Aldrich Inc., St. Louis, MO, USA) was used as a loading control. The Western blot was visualized with supersignal chemiluminescent substrate (Thermo Fisher Scientific), and band intensities were quantified by densitometry analysis (ProteinSimple, Santa Clara, CA, USA).

### Pharmacological inhibitors

Monolayers of astrocytes were treated with 2× the final dose of the MAPK-selective inhibitors, SB203580 (p38K-20 μM), SB202190 (p38K-20 μM), U0126 (ERK1/2-20 μM) and PD184352 (ERK1/2-20 μM) for 1 h before adding equal volume of 2× the final dose of IL-1β treatment.

### Immunocytochemistry

Astrocytes were cultured as adherent monolayers in a 48-well plate at a density of 0.1 × 10^6^ cells per well for 24 h, and then with or without MAPK-selective inhibitors (SB203580, U0126) for 1 h and then inhibitor plus IL-1β (20 ng/ml) for 24 h. Experimental cells were fixed with cold acetone:methanol (1:1) for 30 min at −20 °C. Cells were then blocked in phosphate-buffered saline (PBS) with 2% bovine serum albumin (BSA) and 0.1% triton X-100 for 1 h at room temperature. Cells were incubated in PBS with 2% BSA and 0.1% triton X-100 plus anti-COX-2 antibody at 1:500 and anti-glial fibrillary acidic protein (GFAP) antibody at 1:600 for 8 h at 4 °C. Cells were washed with PBS and then incubated in secondary antibody (1:800) for 1 h at room temperature. Micrographs were taken on a Nikon Eclipse T_*i*_. (Nikon Inc., Melville, NY, USA).

### Statistical analyses

Statistical analyses were carried out using GraphPad Prism 5.0 software (GraphPad Software, Inc., La Jolla, CA), with one-way ANOVA and Newman-Keuls post-test for multiple comparisons. Data generated from each assay from the TaqMan® Human Inflammation Array (Life Technologies, C/N: 4414074) were analyzed independently. Significance was set at *p* < 0.05, and data represent mean values ± SEM. Data presented are representative of a minimum of three independent experiments with two or more independent donors, unless noted, in which case, *n* represents cumulative data from a specific number of independent human donors (TaqMan® Human Inflammation Array and western blots).

## Results

### Human astrocyte IL-1β-induced C/EBPβ, directly or indirectly, regulates 17 of 29 selected astrocyte inflammation genes

As previously reported, IL-1β induces astrocyte C/EBPβ expression and localization to nuclei, where the transcription factor regulates gene expression [7,17]. Astrogliosis is a hallmark of many CNS diseases, yet little is known about how astrocyte C/EBPβ-regulated gene expression may contribute to progression of these pathologies. Here, we used the TaqMan**®** Human Inflammation Array to evaluate human astrocyte C/EBPβ’s contribution to expression of 92 inflammatory genes in response to IL-1β. Figure 1 shows cumulative data from two independent astrocyte donors. Primary human astrocyte C/EBPβ expression was silenced using siRNA technology, and cells were cultured in the presence of IL-1β for 12 h. As Figure 1 indicates, IL-1β altered mRNA levels of 29 of the 92 genes by two-fold or greater. C/EBPβ knockdown by siRNA affected expression of 17 of the 29 genes by 25% or more. Moreover, our data are supported by previous reports, and we confirmed two targets in additional donors. Data from previous studies support our findings that IL-1β-activated astrocytes express higher levels of NOS-2 and intercellular adhesion molecule (ICAM)-1, and each was down- and upregulated, respectively, in C/EBPβ-deficient astrocytes [25,26]. Interestingly, only 4 of the 17 IL-1β-induced genes affected by C/EBPβ are downregulated in C/EBPβ-deficient astrocytes; the remaining 13 genes are upregulated. IL-1β induced the expression of astrocyte prostaglandin endoperoxide synthase 2, or COX-2, mRNA by an average of 824 fold, while C/EBPβ knockdown in parallel experiments led to an average of 37% reduction. IL-1β induced the expression of BDKRB2 mRNA by an average of 35 fold; C/EBPβ knockdown further enhanced this increase by an average of 68%. These data suggest that IL-1β-mediated astrocyte C/EBPβ expression functions to activate or inhibit 17 of 29 of the IL-1β-induced human astrocyte inflammation genes.

### siRNA knockdown of C/EBPβ affects IL-1β-induced astrocyte COX-2 and BRKRB2 expression

**Figure 2:**
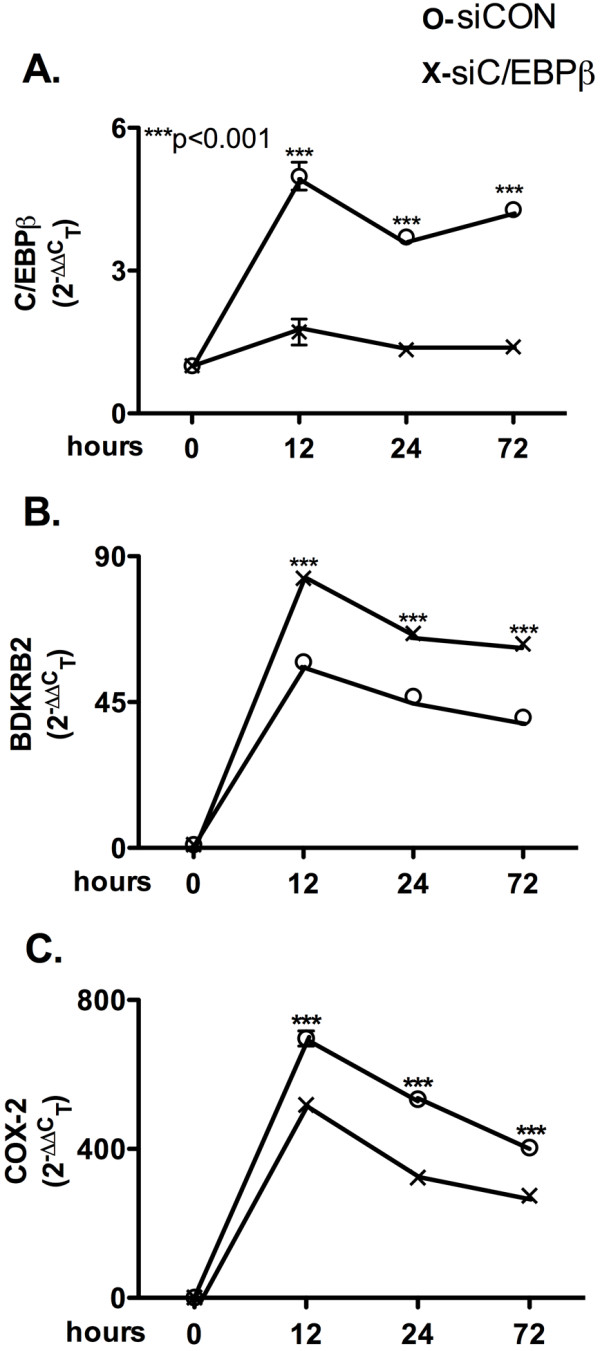
**C/EBPβ knockdown enhances BDKRB2 and reduces COX-2 mRNA.** Astrocytes were transfected with siRNA specific for C/EBPβ or siCON (100 nM), and then cultured for 24 h before being treated with IL-1β (20 ng/ml) for 12, 24 or 72 h. Total RNA was isolated, reverse-transcribed and then assayed by RT^2^PCR for (**A**) C/EBPβ, (**B**) BDKRB2 and (**C**) COX-2 relative transcripts levels. GAPDH was used as the normalizing control; *p*-values are compared to control unless denoted by lines to specific comparisons.

Differences in genetic background among human astrocyte donors account for variation in readouts; therefore, we confirmed our results for COX-2 and BDKRB2 mRNA in two additional astrocyte donors. Consistent with our previously published work [7], a single bolus of IL-1β induced a five-fold increase in astrocyte C/EBPβ mRNA expression at 12 h and maintained a four-fold increase through 72 h (Figure 2A; *p* < 0.001). C/EBPβ-specific siRNA transfection achieved a 65% knockdown through 72 h in IL-1β-treated astrocytes (Figure 2A; *p* < 0.001). We have previously reported that C/EBPβ-specific siRNA alone reduces basal levels of C/EBBβ mRNA by 65% [7]. IL-1β induced a 55-fold increase in astrocyte BDKRB2 mRNA expression at 12 h and maintained increases of 45- (24 h) and 40-fold (72 h) (Figure 2B; *p* < 0.001). C/EBPβ-deficient astrocytes expressed BDKRB2 mRNA levels at 83 (12 h), 65 (24 h) and 60 fold (72 h) that of control siRNA (siCON)-transfected astrocytes (Figure 2B*p* < 0.001). IL-1β induced 700- (12 h), 533- (24 h) and 400-fold (72 h) increases in astrocyte COX-2 mRNA expression, while C/EBPβ knockdown downregulated this robust induction by 26% (12 h), 39% (24 h) and 31% (72 h) (Figure 2C*p* < 0.001). These data confirm the mRNA expression results from the TaqMan**®** Human Inflammation Array plate.

**Figure 3:**
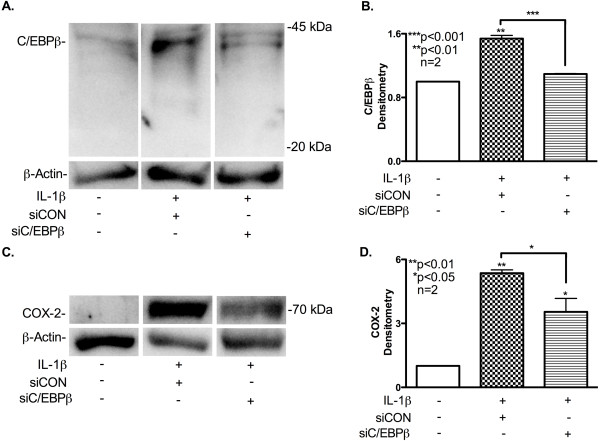
**C/EBPβ knockdown blocks IL-1β-induced C/EBPβ and COX-2 protein levels.** Astrocytes were transfected with siRNA specific for C/EBPβ or siCON (100 nM) and then cultured for 24 h before being treated with IL-1β (20 ng/ml) for 24 h. (**A and C**) Protein lysates were isolated, resolved by SDS-PAGE, transferred to a PVDF membrane and then immunoblotted for C/EBPβ and COX-2 expression. (**B and D**) The densitometry graph is cumulative data from two independent astrocyte donors (*n* = 2) that were normalized to β-actin (**B and D**); *p*-values are compared to control unless denoted by lines to specific comparisons.

Next, we investigated the changes in COX-2 expression by immunoblot analyses in the context of C/EBPβ-specific siRNA transfection followed by IL-1β activation. To confirm siC/EBPβ effectively blocked IL-1β-induced astrocyte C/EBPβ, we performed immunoblot analysis of 24 h protein lysates; results from 72-h lysates were previously reported [7]. IL-1β induced astrocyte C/EBPβ protein expression at 24 h post-treatment; however, transfection with siC/EBPβ reduced these levels (Figure 3A). Densitometry analysis of two independent donors indicates C/EBPβ levels are significantly reduced in siC/EBPβ-transfected astrocytes (Figure 3B*p* < 0.001). IL-1β induced astrocyte COX-2 protein expression at 24 h post-treatment (Figure 3C). IL-1β-induced astrocyte COX-2 was decreased in siC/EBPβ-transfected cells compared to siCON-transfected cells. Densitometry analyses showed significantly more COX-2 protein was expressed in IL-1β-treated astrocytes compared to untreated cells (*p* < 0.01); however, siC/EBPβ-transfected cells expressed reduced COX-2 compared to siCON-transfected cells (Figure 3D*p* < 0.05). These data suggest that C/EBPβ regulates mRNA and protein expression of multiple human astrocyte inflammation genes.

### Differential roles of p38K and ERK1/2 signaling pathways in astrocyte COX-2 and BDKRB2 regulation

**Figure 4:**
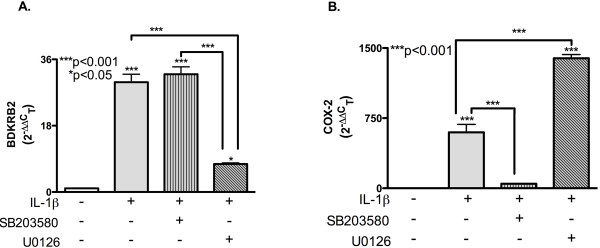
**IL-1β signals through ERK1/2 and p38K to increase astrocyte BDKRB2 and COX-2 mRNA, respectively.** Astrocytes were cultured for 24 h, pretreated with pathway-selective inhibitors (20 μM) and then treated with IL-1β for 12 h. Total RNA was isolated, reverse-transcribed and then assayed by RT^2^PCR for (**A**) BDKRB2 or (**B**) COX-2 relative transcript levels. GAPDH was used as the normalizing control; *p*-values are compared to control unless denoted by lines to specific comparisons.

IL-1β activates a cascade of signal transduction molecules to regulate astrocyte gene expression. Recent studies suggest that IL-1β signals through the p38K pathway to activate C/EBPβ [21]. To determine if a common pathway regulates C/EBPβ and COX-2, and/or if an alternate pathway regulates BDKRB2, astrocytes were treated with SB203580 or U0126, and then with IL-1β for 12 h. Consistent with data from the two donors used for the array plate (Figure 1), IL-1β induced a 30-fold increase in BDKRB2 mRNA compared to untreated cells (Figure 4A*p* < 0.001); pretreating the cells with SB203580 had no significant effect on BDKRB2 mRNA levels compared to IL-1β alone. However, pretreating astrocytes with U0126 significantly reduced the IL-1β-mediated increase in BDKRB2 mRNA by 75% (Figure 4A*p* < 0.001). IL-1β induced a 600-fold increase in COX-2 mRNA compared to untreated cells (*p* < 0.001); SB203580 pretreatment reduced this response by 93% (Figure 4B*p* < 0.001). IL-1β induced a 1,400-fold increase in COX-2 mRNA in U0126-pretreated astrocytes; this was a significant increase compared to astrocytes treated with IL-1β alone (Figure 4B*p* < 0.001). Similar results were obtained using the p38K- and ERK1/2-selective inhibitors SB202190 and PD184352, respectively (Additional file 1). Moreover, these data suggest that the p38K and ERK1/2 pathways are essential for IL-1β-mediated increases in COX-2 and BDKRB2 expression, respectively.

**Figure 5:**
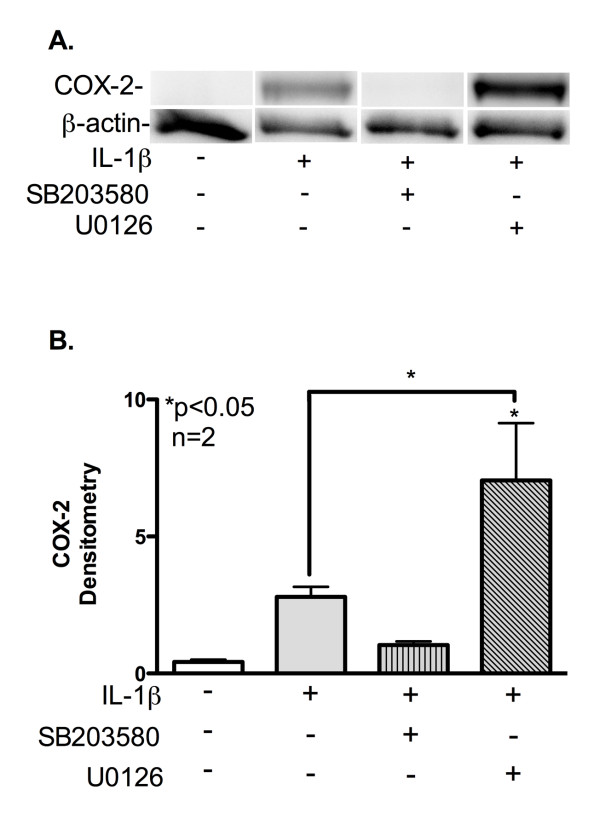
**IL-1β signals through p38K to increase astrocyte COX-2 protein.** (**A-B**) Astrocytes were cultured for 24 h, pretreated with pathway-selective inhibitors (SB203580-20 μM and U0126-20 μM) and then treated with IL-1β for 24 h. Total protein lysates were isolated, resolved by SDS-PAGE, transferred to a PVDF membrane and then immunoblotted for COX-2 expression. (**B**) COX-2 and β-actin bands were analyzed by densitometry analysis, and their intensities graphed using β-actin as the loading control; *p*-values are compared to control unless denoted by lines to specific comparisons. Densitometry fold change data are cumulative from two independent astrocyte donors (*n* = 2).

To confirm that changes in COX-2 and BDKRB2 mRNA lead to changes in protein levels, we pretreated astrocytes with SB203580 or U0126, and then with IL-1β for 24 h. Western blot analysis results were consistent with the mRNA expression studies. COX-2 (70 kDa) was undetectable in total protein lysates from untreated astrocytes; however, a 70-kDa isoform was detected in lysates from IL-1β-treated astrocytes (Figure 5A). The COX-2 signal was undetectable in lysates from SB20380-pretreated astrocytes compared with IL-1β alone (Figure 5A). The COX-2 signal was more intense in lysates from U0126-pretreated astrocytes compared to all other conditions (Figure 5A). Densitometry analyses of the bands revealed higher COX-2 levels in IL-1β-treated astrocytes; however, SB203580 pretreatment blocked this increase (Figure 5B). Significantly more COX-2 was detected from lysates of U0126-pretreated astrocytes compared to untreated and SB203580/IL-1β-treated astrocytes (*p* < 0.05; Figure 5B).

**Figure 6:**
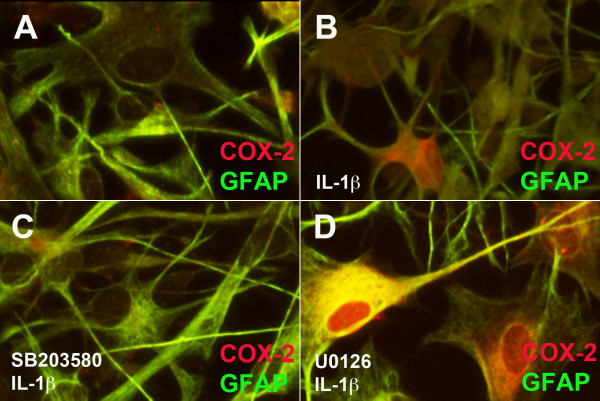
**p38K-selective small molecule inhibitor blocks IL-1β-induced COX-2 expression.** (**A-D)** Astrocytes were cultured in 48-well plates for 24 h, treated with selective inhibitors (SB203580-20 μM, and U0126-20 μM) for 1 h and then IL-1β (20 ng/ml) for 24 h. Cells were fixed, blocked and then incubated with antibodies against GFAP (green) and COX-2 (red). All pictures were taken at ×200, and data represent at least three independent experiments in at least two independent donors.

To determine if blocking p38K or ERK1/2 activity affects IL-1β-mediated COX-2 cellular localization, we pretreated astrocytes with selective inhibitors and then with IL-1β for 24 h, fixed and colocalized GFAP as an astrocyte-specific marker (green) with COX-2 (red, Figure 6A-D) in human astrocytes. The cell body of control cells is large, and the processes are wide (Figure 6A). The processes of activated astrocytes are more condensed, and staining of GFAP is more intense (Figure 6B-D). Low levels of COX-2 are detected in control human astrocytes (Figure 6A) compared with activated astrocytes, where the red signal throughout the cell is enhanced (Figure 6B). Together with the previously shown mRNA and protein expression data, this confirms that COX-2 expression is increased in astrocytes (Figures 2, 3, 4 and 5). Low levels of COX-2 are detected in SB203580-pretreated astrocytes compared to those treated with IL-1β alone (Figure 6C), while the COX-2 signal is enhanced in U0126-pretreated astrocytes (Figure 6D). These data illustrate that SB203580 blocks IL-1β-mediated COX-2 expression, whereas, U0126 enhances IL-1β-mediated COX-2 expression.

## Discussion

Astrocytes are multifunctional glial cells that maintain CNS homeostasis, neuronal signaling, BBB and responses to trauma. Neuroinflammation is a contributing factor of many CNS diseases and profoundly affects astrocyte gene expression [1,27]. Immune-induced changes in astrocyte gene expression are well documented and play an important role in restoring normal CNS function after trauma [1,28]. Currently, investigators lack a full understanding of how astrocytes contribute to the initiation and control of CNS immune responses. To this end, we sought to characterize the role of C/EBPβ in regulating IL-1β-mediated increases in primary human astrocyte expression of a panel of inflammatory genes. Here, we used an array of 92 human inflammatory genes to assay the effect of IL-1β on expression of these genes in two independent human astrocytes donors. Expression of 29 of the 92 mRNAs was affected by at least two fold, and C/EBPβ knockdown affected expression of 17 of the 29 genes by at least 25%. We confirmed IL-1β-mediated COX-2 and BDKRB2 expression, with and without C/EBPβ knockdown. C/EBPβ knockdown decreased COX-2 mRNA and protein levels, while it increased BDKRB2 mRNA expression. Data from a related study show p38K inhibition blocks IL-1β-mediated astrocyte C/EBPβ expression, whereas ERK1/2 inhibition enhances expression [8]. Accordingly, we found that the IL-1β-mediated increase in COX-2 expression is p38K-dependent, whereas IL-1β-mediated expression of BDKRB2 is ERK1/2-dependent. Interestingly, ERK1/2 pathway inhibition exacerbated IL-1β-mediated COX-2 induction. On the contrary, BDKRB2 induction by IL-1β was robustly diminished with ERK1/2 inhibition. Lastly, data showed that IL-1β signals through the p38K pathway to increase expression of COX-2. Our data show that C/EBPβ, in concert with other factors, may contribute to regulation of many human astrocyte genes during neuroinflammation.

Advances in gene expression technology have facilitated the study of astrocytes in disease processes. Genomic array data of IL-1β-induced astrocyte gene expression studies were compiled and thoroughly reviewed by John et al. (2005); several of our data are corroborated therein. As in this report, multiple arrays have shown IL-1β induces CD40, NOS-2, vascular cell adhesion molecule-1, ICAM-1, TNF and COX-2 [29,30]. Other studies have utilized more diverse stimuli to study immune-induced astrocyte gene expression, such as interferon, HIV-1 virion particles or viral proteins [29,30]. Interestingly, HIV-1-treated murine astrocytes increase expression of many of these same genes: CD40, COX-2 and TIMP-1 [30]. Overall, the results of this study corroborate those of past studies while adding critical regulators of neuroinflammation and BBB integrity, such as BDKRB2, to the list of IL-1β-induced human astrocyte genes.

C/EBPβ expression is detectable in immune-activated rodent glia, and it is now clear that this factor is involved in inflammatory processes in tissues throughout the body [17,31]. In the brain, data suggest that C/EBPβ is a direct downstream target of neural growth factor during neurogenesis [32]. C/EBPβ activates regeneration-associated gene expression following axon injury and provides cerebral protection to excitotoxic injury in mouse brains [33-35]. Our group recently reported that C/EBPβ is detectable in brains of HIV-1-infected patients and the factor contributes to regulating IL-1β-mediated astrocyte TIMP-1 [7]. C/EBPβ regulates IL-1β-mediated human astrocyte C3 expression [21], but more in-depth studies on human astrocytes are lacking [20,21]. Here, we found that the majority of IL-1β-induced transcript levels were affected by C/EBPβ knockdown. Studies have shown that all three isoforms can function as repressors of transcription [16]. C/EBPβ binds with C/EBPδ, nuclear factor (NF)κB and activator protein-1 (AP-1) in various cell types to affect gene expression [36]. NFκB is a key factor in IL-1β-induced gene expression; however, varying combinations of transcription factors may determine how transcription is affected at each promoter. Furthermore, posttranslational modifications can affect transcription factor function; ERK1/2-mediated phosphorylation or sumoylation represses C/EBPβ transcription [16,37]. Therefore, manipulating C/EBPβ expression levels alone may have limited effect. Conversely, overexpression or repression of multiple factors or mutations that alter their posttranslational modifications may provide a route to modify gene expression in a highly specific manner. To this end, researchers must identify the proportion, and derivatives of the various important factors (NFκB, C/EBPβ, C/EBPδ and AP-1), and then manipulate them in a way that results in therapeutic changes in gene expression. Such drug formulations would have the potential to limit side effects while maximizing potency. Here, C/EBPβ knockdown increased expression of 13 of 17 transcripts tested.

As previously reported, NOS-2 and ICAM-1 expression levels were negatively and positively affected by C/EBPβ knockdown, respectively [15,19]. Of the four mRNA levels decreased in siC/EBPβ-transfected astrocytes, we first chose to focus on COX-2, the most highly induced gene that was affected by C/EBPβ knockdown. COX-2 is an enzyme that converts arachidonic acid to prostaglandin endoperoxide H2. COX-2 enzyme is a key player during inflammation and therapeutic target of non-steroidal anti-inflammatory drugs. C/EBPβ affects COX-2 expression in a cell-dependent manner; supporting the notion that multiple factors and the derivatives thereof ultimately determine gene expression in any given cell type [16,36]. The regulation of COX-2 via C/EBPβ is thus highly significant both from the perspective of understanding inflammation as well as therapeutic approaches. C/EBPβ, C/EBPδ, NFκB, cyclic adenosine monophosphate response element-binding protein and AP-1 all regulate COX-2 expression in various cell types [15,38]. C/EBPβ is essential for rodent macrophage biphasic expression of COX-2; however, in A431 cells, all C/EBPβ isoforms repress COX-2 expression, whereas fibroblast COX-2 expression is not C/EBPβ-dependent [16,36,39]. These data suggest that the highly inducible COX-2 may be regulated in a cell-specific manner to respond to inflammatory stimuli. In our studies, IL-1β induced robust increases in human astrocyte COX-2 expression; COX-2 was the second most induced gene among all donors tested. Additionally, p38K signaling and C/EBPβ expression may be crucial for astrocyte COX-2 expression during neuroinflammation. In murine macrophages, IL-1β signals through ERK1/2 to increase COX-2 expression [36]. In our studies, an ERK1/2-selective inhibitor increased IL-1β-mediated human astrocyte C/EBPβ and COX-2 expression [7]. MAPK pathway activity is implicated in many disease processes, and therefore, pathway-selective inhibitors are being tested as effective therapies [40]. The current studies illustrate why these pharmacological inhibitors may represent a “blunt-ended” tool when trying to affect expression-specific genes. Blocking a specific pathway or even transcription factor can have profound effects; however, manipulating multiple factors in specific ways may allow fine-tuning of gene expression. These data suggest that the ERK1/2 pathway may activate inhibitors of C/EBPβ and thereby inhibit COX-2 expression. Indeed, ERK1/2 is capable of phosphorylating and thereby repressing C/EBPβ activity [37].

C/EBPβ knockdown increased IL-1β-induced astrocyte BDKRB1 and BDKRB2 expression as well as that of 11 other genes (Figure 1). We chose to further investigate BDKRB2 expression because of the key role the kallikrein-kinin system plays in the CNS [41]. Most of the biological effects of the kinin system are mediated through BDKRB2 [41]. Interestingly, BDKRB2 signaling mediates prostaglandin release [42]; collectively, these data suggest that C/EBPβ may play a key role in modulating the inflammatory response by effectively decreasing BDKRB2 signaling (through downregulation of mRNA) while concomitantly increasing COX-2 expression. These data bolster the assertion that transcription factors may function in a context-dependent manner, possibly even within a single cell. Data suggest BDKRB2 signaling may increase BBB permeability [43-46]; an important function during CNS immune responses [47]. Our group is particularly interested in the progression of HAD, and BBB permeability is a contributing factor to HIV-1 entering the CNS [48,49]. IL-1β induced a robust increase in human astrocyte BDKRB2 mRNA expression, and C/EBPβ knockdown consistently enhanced this effect. Furthermore, ERK1/2 signaling is critical for IL-1β-mediated astrocyte BDKRB2 expression. The p38K-selective inhibitor showed an increase, but no significant effect on BDKRB2 mRNA levels. These signaling data further support complementary regulation of astrocyte COX-2 and BDKRB2 at the level of transcription factors and signal transduction. These data may lead to novel mechanisms to manipulate prostaglandin production and affect inflammation in tissue microenvironments.

## Conclusions

**Figure 7:**
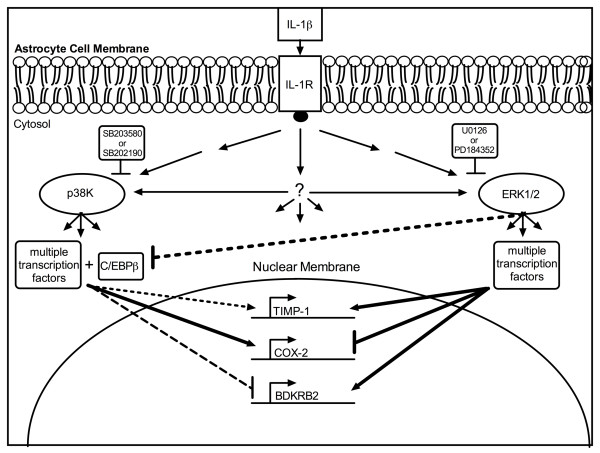
**IL-1β activates astrocyte MAPK pathways, activates transcription factors and thereby regulates multiple human inflammation genes.** IL-1β-mediated activation of astrocyte MAPK [p38K and ERK1/2] pathways precedes robust changes in gene expression. Multiple transcription factors traffic to astrocyte nuclei where they facilitate changes in mRNA transcription. Blocking IL-1β-mediated activation of astrocyte p38K blocks C/EBPβ translocation to nuclei [8]. The subsequent effects are similar to C/EBPβ knockdown; IL-1β-induced astrocyte TIMP-1 and COX-2 expression is blocked, and BDKRB2 expression is enhanced (Figure 4) [8]. ERK1/2 inhibition completely blocks IL-1β-induced astrocyte BDKRB2 expression and TIMP-1 expression, but enhances IL-1β-induced C/EBPβ and COX-2 expression (Figures 4,5 and 6) [8]. Two scenarios involving C/EBPβ could explain these events: (1) Blocking ERK1/2 signaling inhibits factors essential for IL-1β-induced astrocyte TIMP-1 expression and results in increased C/EBPβ translocation to nuclei to compensate for reduced TIMP-1 transcription. Increased C/EBPβ may account for increased COX-2. (2) Alternatively, ERK1/2 signaling may directly down-modulate IL-1β-induced astrocyte C/EBPβ; therefore, ERK1/2-selective inhibitors may increase COX-2 through an increase in CEBPβ activity. In either scenario, ERK1/2 is essential, and C/EBPβ is an auxiliary path to IL-1β-induced astrocyte TIMP-1. These data suggest MAPK, p38K and ERK1/2 are part of an intricate regulation network in which the two kinases balance one another’s activity to achieve a graded astrocyte response to CNS injury.

Overall, with the data in the current study and other ongoing investigations, we shed some light on the complex nature of astrocyte gene regulation during neuroinflammation. Astrocyte C/EBPβ, TIMP-1, COX-2 and BDKRB2 regulation show that p38K and ERK1/2 may differentially regulate multiple genes in a graded manner. Furthermore, C/EBPβ may act to repress some ERK1/2-regulated genes and activate p38K-regulated genes while acting in a more auxiliary role regulating genes such as TIMP-1 (Figure 7). As with all expression array readouts, the gene targets need to be individually confirmed *in vitro*, *in vivo* and then in disease. Nonetheless, in addition to identifying new genes affected by C/EBPβ knockdown, this work illustrates the complexity of astrocyte gene regulation and the need to outline species- and cell-type-specific regulation of important inflammatory mediators. Our findings help to identify and understand the transcriptome of factors that mediate human astrocyte inflammatory response. Identifying the other factors that join with C/EBPβ to regulate human astrocyte inflammatory responses may provide new therapeutic targets for ameliorating CNS pathology.

## Abbreviations

AP, activator protein; ANOVA, analysis of variance; BBB, blood–brain barrier; BSA, bovine serum albumin; BDKRB2, bradykinin receptor b2; C/EBP, CCAAT enhancer-binding protein; CNS, central nervous system; COX, cyclooxygenase; ERK, extracellular regulated kinase; GAPDH, glyceraldehyde phosphate dehydrogenase; GFAP, glial fibrillary acidic protein; HAD, HIV-1-associated dementia; HIV, human immunodeficiency virus; ICAM, intercellular adhesion molecule; IL, interleukin; kDa, kilodalton; NFκB, nuclear factor kappa B; NOS, nitric oxide synthase; p38K, p38 kinase; PBS, phosphate-buffered saline; PVDF, polyvinyl difluoride; RT2PCR, real-time reverse transcription polymerase chain reaction; si, small interfering; SDS-PAGE, sodium dodecyl sulfate polyacrylamide gel electrophoresis; TIMP-1, tissue inhibitor metalloproteinases-1; TNF, tumor necrosis factor.

## Competing interests

The authors have no financial or non-financial competing interests.

## Authors’ contributions

JF participated in the conception, design and execution of all experiments, and carried out data analysis and constructed the manuscript. AG participated in the conception, design and trouble-shooting of all experiments, and was essential in the design and development of the manuscript. Both authors read and approved the final manuscript.

## Authors’ information

JF is a fifth year graduate student; this work, together with a concomitantly submitted manuscript, represents the completion of JF’s dissertation project. AG is JF’s graduate mentor/advisor.

## Supplementary Material

Additional file 1 IL-1β signals through ERK1/2 and p38K to increase astrocyte BDKRB2 and COX-2 mRNA, respectively. (**A**-**B**) Astrocytes were cultured for 24 h, pretreated with pathway selective inhibitors (20 μM) and then treated with IL-1β for 12 h. Total RNA was isolated, reverse-transcribed and then assayed by RT^2^PCR for (**A**) BDKRB2 or (**B**) COX-2 relative transcript levels. GAPDH was used as the normalizing control; *p*-values are compared to control unless denoted by lines to specific comparisons. Click here for file
