# Supplementary figures and images for: C/EBPβ regulates multiple IL-1β-induced human astrocyte inflammatory genes
Source: J Neuroinflammation. 2012 Jul 20;9:177. doi: 10.1186/1742-2094-9-177 (PMC3464795; doi:10.1186/1742-2094-9-177)

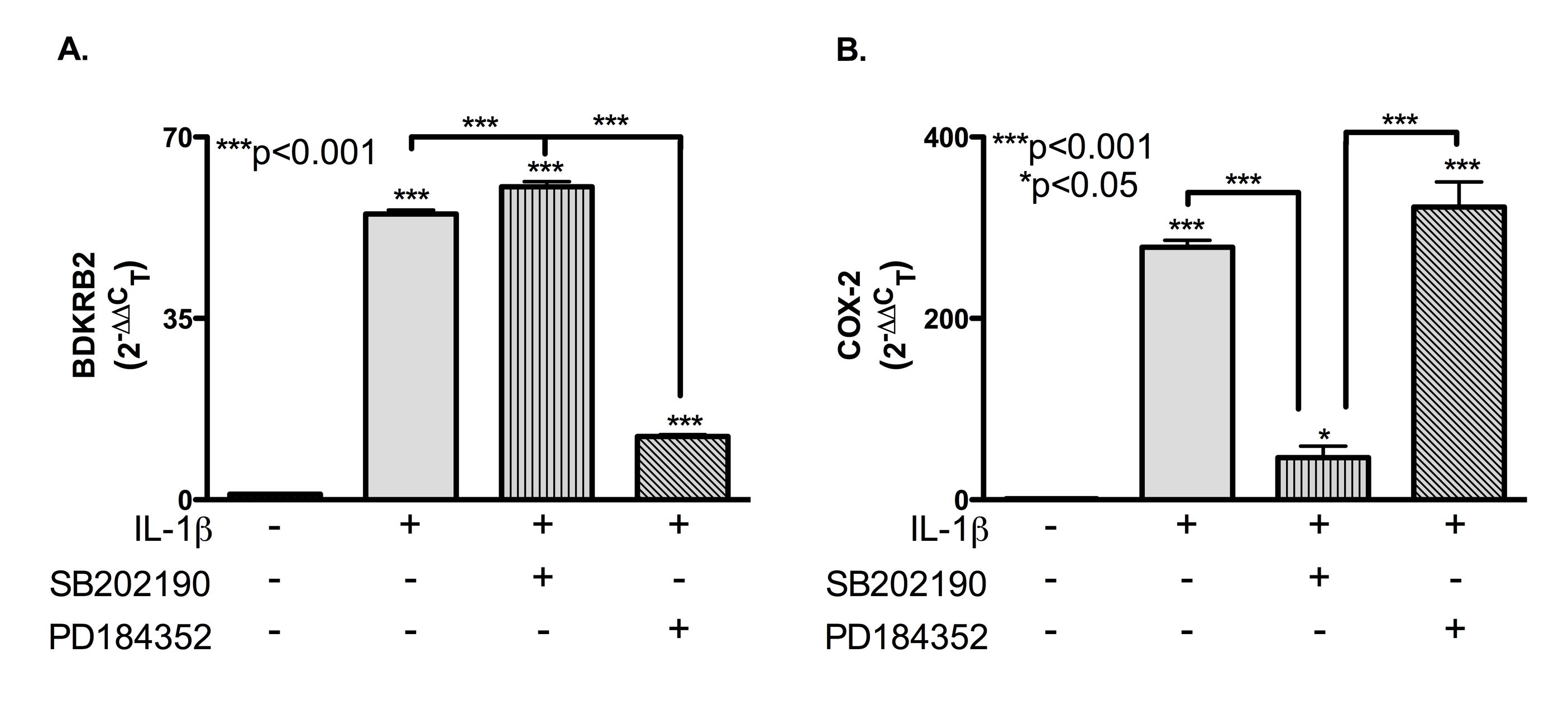

Supplement: Additional file 1 — IL-1β signals through ERK1/2 and p38K to increase astrocyte BDKRB2 and COX-2 mRNA, respectively. (A-B) Astrocytes were cultured for 24 h, pretreated with pathway selective inhibitors (20 μM) and then treated with IL-1β for 12 h. Total RNA was isolated, reverse-transcribed and then assayed by RT2PCR for (A) BDKRB2 or (B) COX-2 relative transcript levels. GAPDH was used as the normalizing control; p-values are compared to control unless denoted by lines to specific comparisons. [file 1742-2094-9-177-S1.tiff]
